# Interesting Case of Contained Perforated Peptic Ulcer With Pancreatic Communication Causing Hemorrhagic Shock

**DOI:** 10.7759/cureus.56992

**Published:** 2024-03-26

**Authors:** Scott Edelson

**Affiliations:** 1 Department of Medicine, Brooke Army Medical Center (BAMC), San Antonio, USA

**Keywords:** acute gastrointestinal bleed, endoscopy, contained perforation, gastrointestinal perforation, complicated peptic ulcer disease

## Abstract

Peptic ulcer disease (PUD) is a common gastrointestinal diagnosis affecting the stomach and proximal duodenum. A contained perforation with pancreatic communication is an exceedingly rare subtype where gastroduodenal perforation is limited by the surrounding pancreas, preventing free leakage of gastric and pancreatic contents into the peritoneal cavity. A 48-year-old male with a history of perforated antral ulcer requiring surgical management and placement of a Graham patch presented with upper gastrointestinal bleeding. Initial esophagogastroduodenoscopy (EGD) showed a new clean-based antral ulcer; however, the patient continued to experience hematemesis post-procedure. A repeat EGD revealed the same antral ulcer now with suture material exposed near the prior site of the Graham patch, along with a soft tissue mass resembling the pancreas and no evidence of active bleeding. Following this EGD, the patient had profuse hematemesis with hemorrhagic shock and underwent emergent exploratory laparotomy confirming contained posterior perforation of the stomach with complete erosion of the stomach wall onto the head of the pancreas. This case highlights an atypical presentation for a perforated peptic ulcer (PPU) with pancreatic communication.

## Introduction

Peptic ulcer disease (PUD) is a common gastrointestinal diagnosis affecting the stomach and proximal duodenum. Gastroduodenal perforations are a rare but serious complication of PUD with a reported mortality rate of up to 27% [1,2]. Common risk factors for PUD and associated perforation include chronic non-steroidal anti-inflammatory drug (NSAID) use, tobacco use, Helicobacter pylori infection, and critical illness. Classically, perforated peptic ulcers (PPU) present with acute-onset, severe epigastric pain, abdominal distention, and vomiting, often with radiographic evidence of free air [3]. A contained perforation with pancreatic communication is an exceedingly rare subtype where the gastroduodenal perforation is limited by the surrounding pancreas, preventing free leakage of gastric and pancreatic contents into the peritoneal cavity [4]. These are often difficult to diagnose due to their varied presentation, which can lead to delayed treatment with increased morbidity and mortality. While reports of contained perforations are limited, presentations can range from minimal epigastric pain to bacterial peritonitis or pancreatitis. Management is typically multidisciplinary, with prompt imaging, initiation of antibiotics, and sometimes surgical intervention [5]. We present an atypical case of a PPU with pancreatic communication in a hemodynamically unstable patient requiring emergent surgical management.

## Case presentation

A 48-year-old male with a past medical history significant for chronic pain with daily NSAID use and tobacco use presented with two days of epigastric pain, hematemesis, and melena. The patient reported a history of a perforated antral ulcer one year prior requiring surgical management and placement of a Graham patch, a portion of omentum used to cover and seal the perforation sutured through the exterior portion of the defect. On initial presentation, he was found to be volume-depleted with an initial blood pressure of 100/75 and a heart rate of 110 beats per minute. He was afebrile and saturating well on room air. Examination revealed a nondistended abdomen with mild epigastric tenderness without rebound or guarding. The remainder of his physical exam was non-contributory. Initial laboratory studies were notable for hemoglobin of 7.26 (baseline 12), white blood cell count (WBC) of 10.72, creatinine of 1.5 (baseline 1.2), blood urea nitrogen (BUN) of 47, lipase of 72, and normal liver associated enzymes (LAEs). A CT abdomen and pelvis with contrast showed normal appearing pancreas without evidence of pneumoperitoneum. The patient was adequately resuscitated and made NPO for esophagogastroduodenoscopy (EGD). EGD revealed a clean-based antral ulcer without high-risk stigmata that did not require any intervention. Of note, this newly discovered ulcer was adjacent to the previously repaired ulcer. The patient remained hospitalized for further observation.

**Figure 1:**
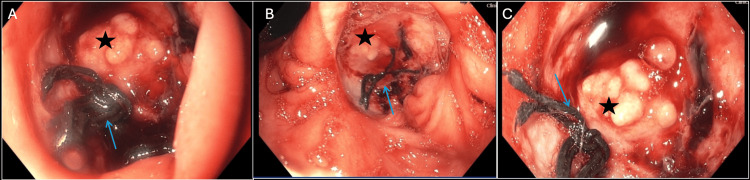
Contained Perforation With Visualization of the Head of the Pancreas Star, head of the pancreas; arrow, Graham patch sutures

Several days later, the patient experienced hematemesis and melena prompting a repeat EGD, which showed the antral ulcer now with suture material near the prior site of the Graham patch, along with a soft tissue mass resembling the pancreas and no evidence of active bleeding. Given that a Graham patch should not be visible from the interior of the stomach, there was a high clinical concern for a contained perforation with pancreatic communication (Figure 1A-1C, the star represents the head of the pancreas and arrow the Graham patch sutures). Surgery was consulted at that time and recommended urgent surgical intervention.

Following EGD, the patient remained afebrile and hemodynamically stable with unchanged serial abdominal examinations. Overnight, the patient had profuse hematemesis and decompensated due to hemorrhagic shock requiring multiple blood transfusions and vasopressor support. CT angiography did not show any active bleeding but revealed focal attenuation involving the gastroduodenal artery indicating the possible source of upper GI bleed. Interventional radiology was consulted, but they were not able to control the hemorrhage through arterial embolization. The patient then underwent emergent exploratory laparotomy confirming contained posterior perforation of the stomach with complete erosion of the stomach wall into the head of the pancreas, with prior Graham patch sutures in the affected area. Simple oversewing of the perforated gastric ulcer was performed successfully. Following surgery, the patient had an uncomplicated recovery and was discharged without further complications.

## Discussion

PUD represents a significant cause of morbidity worldwide. PPU is a serious complication that requires urgent evaluation and treatment by a multidisciplinary team including surgical and gastrointestinal specialists. PPU often presents with sudden onset, severe epigastric pain, abdominal distention, and vomiting [3]. However, contained PPU can be much more difficult to diagnose. Contained perforations prevent gastric contents from leaking into the peritoneum and causing peritonitis. Therefore, the classic triad of symptoms in PPU is not typically present in patients with contained perforations. This case highlights the difficulty of identifying contained perforation with pancreatic communication. Without radiographic evidence or high clinical suspicion of perforation, pancreatic tissue filling a PPU may have the appearance of a clean-based ulcer. In this case report, the final diagnostic clue of perforation was the visualization of intraluminal Graham patch suture material.

While there is an abundance of literature describing the epidemiology and clinical presentation of acute, free PPU, there is limited information on the incidence and presenting signs and symptoms of contained perforations. According to limited data on contained PPU, the most common presenting symptoms are chronic epigastric pain with radiation to the back, nocturnal pain, and change in severity, location, or radiation of abdominal pain [6]. A comprehensive literature review conducted specifically on gastric ulcer perforation with pancreatic communication found limited reports. One case report described a gastric perforation with pancreatic communication presenting as acute pancreatitis with an elevated lipase and CT findings suggestive of pancreatitis as well as melena in a hemodynamically stable patient [7]. An additional report in the American College of Gastroenterology Case Reports Journal demonstrated a PPU with pancreatic communication in a stable patient complaining of epigastric pain without signs of pancreatitis or peritonitis [8]. In this case, we report a perforated gastric ulcer with pancreatic communication presenting as shock secondary to gastrointestinal hemorrhage.

This case not only emphasizes the challenges of diagnosing contained antral perforations with pancreatic communication but also the severe complications of this rare presentation. Despite the clean-based ulcer without high-risk stigmata appearance of the lesion, a history of previous gastric surgeries or the location alone should prompt further evaluation of a possible perforation. A multidisciplinary approach involving imaging and other specialties is paramount to avoid further complications such as pancreatitis, peritonitis, or abscess formation. This case highlights the importance of maintaining a high index of clinical suspicion for atypical diagnoses in patients with gastrointestinal symptoms, especially in the context of posterior antral peptic ulcers. The key point is that regardless of initial presentation, providers must continually reassess their patients, consider alternative diagnoses, and repeat workups or imaging when the clinical course shifts.

## Conclusions

Peptic ulcers are a common etiology of gastrointestinal hemorrhage. Perforations are a feared complication with significant morbidity and mortality. This case report highlights diagnostic challenges in a patient with an atypical presentation for pancreatic involvement in gastric perforation. It emphasizes the importance of having a high clinical suspicion of perforation in patients with antral ulcers and a history of surgical procedures. A multidisciplinary approach involving imaging, antibiotics, and, in some cases, surgical intervention is crucial for achieving optimal outcomes in such cases.
